# Non-Alcoholic Fatty Liver Disease in Children

**DOI:** 10.3390/medicina57070719

**Published:** 2021-07-16

**Authors:** Jernej Brecelj, Rok Orel

**Affiliations:** 1 Department of Gastroenterology, Hepatology and Nutrition, University Children’s Hospital Ljubljana, Bohoriceva 20, SI-1000 Ljubljana, Slovenia; rok.orel@kclj.si; 2 Department of Paediatrics, Faculty of Medicine, University of Ljubljana, Bohoriceva 20, SI-1000 Ljubljana, Slovenia

**Keywords:** pediatric fatty liver disease, obesity, lifestyle modification, diet, physical activity

## Abstract

*Background and Objectives*: The prevalence of pediatric non-alcoholic fatty liver disease is increasing. A lot of new data are published regularly. *Materials and Methods*: Original clinical studies, review articles, and guidelines in children were searched for and the most relevant included in this review. *Results*: A total of 138 retrieved papers were classified into pathogenesis, epidemiology, diagnosis, and treatment. Pathogenesis is currently explained with the “multi hit hypothesis”, with complex interactions of genetic and environmental factors which trigger inflammation in steatotic liver. The prevalence is rising. A diagnosis can be made with laboratory tests, imaging, and liver biopsy after the exclusion of other causes of liver steatosis. The mainstay of treatment is lifestyle modification consisting of dietary intervention and increased physical activity. The progression to liver cirrhosis can occur even in children. *Conclusions*: Non-alcoholic fatty liver disease in children is a part of a metabolic syndrome in the majority of patients. Due to its complex etiology and high prevalence, multidisciplinary teams, together with public health professionals, should be involved in its treatment.

## 1. Introduction

Pediatric non-alcoholic fatty liver disease (NAFLD) is the most common chronic liver disease in children, with its prevalence rising in parallel with the increased rates of overweight and obesity. NAFLD is a multisystem disease also affecting extrahepatic organs, and it has a long-term impact on health which extends into adulthood and causes significant morbidity and mortality [1,2]. 

The criterion for diagnosis is ≥5% of hepatocytes with macrovesicular steatosis with no evidence of viral, autoimmune, inherited metabolic, or drug-induced liver diseases in a person without excessive alcohol intake [1]. NAFLD results from a combination of genetic impact and epigenetic influences from the prenatal and postnatal period with a strong psychosocial impact [3,4]. 

An international panel of hepatologists proposed a different terminology to avoid the term non-alcoholic, which is sometimes difficult to assess in adults and is inappropriate in children: metabolic dysfunction associated fatty liver disease (MAFLD), instead of NAFLD. New diagnostic criteria were defined: hepatic steatosis detected by imaging, blood biomarkers or liver histology in overweight/obese persons or those with type 2 diabetes mellitus, or lean/normal weight persons with additional metabolic risks, such as high triglycerides or low high density cholesterol, glucose intolerance, increased waist circumference, and increased blood pressure [5]. However, the same definition and nomenclature is not appropriate for the population of children as it is too narrow and does not take into consideration inherited metabolic diseases [6,7]. A more appropriate term proposed especially for young children is pediatric fatty liver disease (PeFLD) [6]. The change in terminology is additionally justified by the recently published review in which the criteria for diagnosis in adolescents are also proposed [8]. As there is not yet a wide international acceptance of this meaningful terminological change, we decided to keep the term NAFLD for the purpose of this review, as it is still mostly used.

NAFLD encompasses a wide spectrum of manifestations, from isolated hepatic steatosis without inflammation, to advanced form non-alcoholic steatohepatitis (NASH) with histologic features of inflammation and fibrosis, which may lead to cirrhosis and to end-stage liver disease [1]. An important consideration is that hepatocellular carcinoma is a possible, although rare, complication that can also develop in non-cirrhotic liver [2].

A lot is already known regarding NAFLD in children and new data are reported daily. The implementation of this knowledge on diagnosis and treatment poses a great challenge, as has been shown in a survey of the majority of practicing pediatric hepatologists in university hospitals in Canada [9]. The diagnosis of NAFLD was made by a combination of elevated transaminases, suggestive imaging, and a negative workup for other liver diseases. Liver biopsy was performed in only 14% of patients. The search for and treatment of comorbidities (i.e., obstructive sleep apnea, insulin resistance) was infrequent. The treatment was lifestyle modification (physical activity and dietary modifications) in all providers; vitamin E has been occasionally prescribed by less than half of providers. Follow-up, both according to the frequency of visits (once or twice yearly) and the investigations performed (laboratory, imaging) was very heterogeneous.

The purpose of this review is to summarize up-to-date and concise information on NAFLD in children and to provide brief information to practicing clinicians on recent advances and approaches to the prevention, diagnosis, and treatment of pediatric patients with NAFLD.

## 2. Materials and Methods

The search strategy was focused on original clinical studies, review articles, and guidelines. 

The online databases searched included PubMed, Embase, and Cochrane. The searches were performed between 25 February and 31 March 2021. Search terms were “non-alcoholic fatty liver disease”, “non-alcoholic steatohepatitis”, “fatty liver”, “metabolic dysfunction associated fatty liver disease”, and “pediatric fatty liver disease”. The search was limited to publications in English, children less than 19 years old, clinical trials, systematic reviews, and guidelines. 

During the first check, the titles, abstracts, and key words were searched and publications not meeting the above search criteria were eliminated.

In the next check, all retrieved articles were classified according to the subcategory (e.g., diagnosis, treatment, etc.), type of article (systematic review and guideline or clinical trial), and publication date. 

For each subcategory, the most relevant systematic review or guideline was selected for inclusion in this review. Relevant control trials, which were not included in the systematic review or guidelines or were published after the search of the systematic review or guidelines had finished, were also selected for inclusion. 

## 3. Results

**Figure 1:**
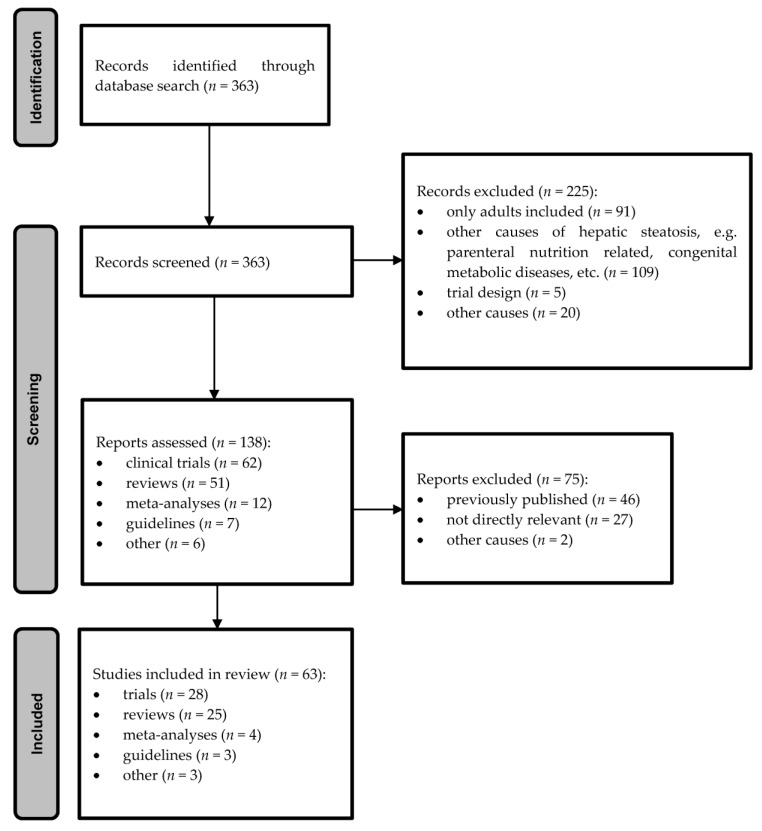
The diagram of search and selection process.

Several searches in accordance with the above criteria revealed 304 clinical trials. Those not fulfilling the search criteria were not considered for further analysis (197 hits) due to various reasons (studies on adults 77, other causes for hepatic steatosis in children and not NAFLD, e.g., parenteral nutrition related, genetic diseases 97, trial designs 4, other 19). The search for guidelines or systematic reviews with the above search criteria revealed 59 hits, of which 13 were excluded as they were for only for adult population, 12 were not on NAFLD but different causes of hepatic steatosis, mostly in connection to parenteral nutrition, one was design, and two were not in English. The diagram of the study search and selection process is shown on Figure 1.

### 3.1. Pathogenic Mechanisms and Risk Factors

The pathogenesis of NAFLD is not yet completely explained, but the “multi hit hypothesis” is currently accepted. “First hit” may represent liver fat accumulation caused by obesity and insulin resistance, which is influenced and maintained by complex interactions of genetic and environmental factors, as well as crosstalk between different organs and tissues, adipose tissue, and the pancreas, gut, and liver [10].

Genes involved in inflammation, lipid metabolism, and oxidation also play an important role and are associated with progressive liver disease, insulin resistance, type 2 diabetes mellitus, and a higher risk for the development of hepatocellular carcinoma [10]. Among several identified, the most studied gene is *PNPLA3.* The carriers of the allele I148M have two-fold higher fat content than non-carriers. Another allele, S453I, which is protective, is found in African Americans and explains why the prevalence of NAFLD in this community is lower than in, e.g., the Hispanic community [10]. 

Prenatal factors predisposing for NAFLD are maternal body mass index, metabolic syndrome, gestational diabetes, and low birth weight of the child [11] influencing the metabolic programming. It explains how prenatal and early postnatal exposures modulate cytogenesis, organogenesis, metabolic and endocrine response, and epigenetic regulation of gene expression. With all these influences, lifelong health and disease risk is programmed in a way that may lead to obesity and insulin resistance, which are risk factors of NAFLD [3]. 

Free sugars, such as sucrose or fructose, are consumed in quantities two to three times the recommended intake, which is less than 10% of energy intake, and cause a fatty liver due to overweight and obesity. Especially high intake of fructose, which is metabolized mostly in the liver, affects hepatic energy metabolism with modulation of the liver gene expression involved in the regulation of different metabolic pathways which lead to hepatic steatosis, with fructose being an inducer of and a substrate for hepatic lipogenesis [12]. A high intake of fructose can explain some cases of “lean” NAFLD [12].

Decreased physical activity is one of the major factors for NAFLD development in overweight or obese children. In a study of 115 overweight/obese children, hepatic fat content measured with magnetic resonance imaging was lower in those with better cardiorespiratory and musculoskeletal fitness as well as speed-agility [13]. The influence of reduced physical activity due to schools closing during the coronavirus disease 2019 epidemic was studied in a cohort of 90 obese children aged 6 to 18 years in South Korea [14]. During a 3 month period of closed schools, they found a significant increase in body weight (from 67.2 ± 23.8 to 71.1 ± 24.2; *p* < 0.001), body mass index (from 26.7 ± 4.6 to 27.7 ± 4.6; *p* < 0.001), and also in AST, ALT, triglycerides, and LDL. 

Often neglected, but of much importance, is adequate sleep. Sleep shortages have been shown to be more prevalent in a group of 67 children and young adults with NAFLD in comparison to controls without NAFLD, probably due to the increased risk of obesity [15,16]. 

Another risk factor for NAFLD is socioeconomic deprivation. It was assessed in a study of two large cohorts of children performed in the United States of America. A group of 334 was diagnosed by magnetic resonance imaging and 245 by liver biopsy. Social deprivation was determined by the Community Deprivation Index which takes into account income, education, public assistance, housing, and insurance status. Socioeconomic deprivation was associated with earlier onset of NAFLD but not with its severity [17].

The psychosocial aspect is also an important part of NAFLD pathogenesis and has to be considered in the management of individual patients and in the planning of health programs combating obesity in children. The psychosocial influence on hepatic steatosis in children is bidirectional. On one hand, children with NAFLD as a consequence of obesity report increased levels of psychological distress, and on the other hand, mental disorders may be associated with the increased risk of obesity and NAFLD [18]. 

Obesity is not a necessary condition for the development of NAFLD. It can be found in 8 to 16% of non-obese children. The causes are visceral obesity in otherwise non-obese children, and genetic and environmental factors [16,19]. Adipose tissue is implicated in endocrine processes, among them the secretion of leptin. Peripheral leptin resistance is linked to the development of NAFLD and metabolic syndrome in children [20]. Liver steatosis and inflammation is influenced by gut microbiota and intestinal permeability, known as the “gut–liver” axis [21]. In a study of 61 pediatric patients with NAFLD or obesity, their stool microbiota studied by metagenomics and metabolomics had different microbiome composition compared to 54 healthy controls. In the stool samples of NAFLD patients, a low abundance of *Oscillospira* and a higher level of 2-butanone was found [22]. The above pathogenetic mechanisms lead to the hepatocyte fat deposits. These increase metabolic activity and possibly cause injury due to the surplus of free radicals. Glutathione is an intracellular tripeptide involved in buffering oxidative stress. It is especially abundant in the liver. Fatty acid accumulation in the liver parenchyma leads to an increased metabolism and reactive oxygen species production, which is at first compensated by higher levels of glutathione, and probably presents the next step in NAFLD progression [23]. 

### 3.2. Epidemiology

The prevalence of NAFLD in children from the general population is 7.6%. In selected populations of obese children, it is present in 34.2%, as was found in a systematic review and meta-analysis of 74 studies [24]. The diagnosis of NAFLD was based on any diagnostic method: liver enzymes, ultrasound, magnetic resonance or other scans, and liver biopsy.

Detailed gender analysis revealed prevalence of NAFLD in 9% (6.5% to 12.5%) of males and in 6.3% (3.8% to 10.4%) of females. In a subgroup analysis according to the nutritional status, 2.3% (1.5% to 3.6%) of those with normal weight, 12.5% (9.2% to 16.7%) of overweight and 36.1% (24.6% to 49.4%) of obese children had NAFLD [24].

A recently published population study of 867 adolescents from the United States of America included in the National Health and Nutrition Examination Survey 2017–2018 found even more alarming data assessing the prevalence of NAFLD with transient elastography: 24.2% of all participants had any degree of steatosis, and 4.4% of them had significant liver fibrosis not associated with either overweight/obesity nor ALT level, but the findings were not confirmed or further analyzed by liver biopsy [25].

### 3.3. Diagnosis

The diagnostic criteria for NAFLD/MAFLD in children, in whom liver steatosis has been diagnosed by biomarkers, imaging, or liver biopsy, are [8,26]:

1. Overweight (body mass index between 85th and 95th percentile) or obesity (body mass index more than 95th percentile), or abdominal obesity (waist circumference of ≥90th percentile for age and gender) or;
2. Fasting plasma glucose of >100 mg/dL (5.6 mmol/L) or known diabetes mellitus type 2 or;
3. In lean children, the presence of at least two metabolic risk disturbances:
  - Elevated triglycerides ≥150 mg/dL (≥1.7 mmol/L);
  - HDL-cholesterol <40 mg/dL (<1.03 mmol/L);
  - Systolic blood pressure ≥130 or diastolic blood pressure ≥85 mmHg;
  - Homeostatic model assessment for insulin resistance (HOMA-IR ≥ 2.5);
  - High-sensitivity C-reactive protein >2 mg/L.

An additional condition is that other causes of fatty liver, such as inborn metabolic diseases, autoimmune and infectious causes are ruled out regardless of obesity or other risk factors for NAFLD [6,8,26,27].

#### 3.3.1. Screening for NAFLD

There is neither an ideal method nor an international consent on whom and with which method to screen. In adult populations, ALT was not a good indicator of liver injury, as one-third of patients with normal ALT already had NASH and significant liver fibrosis, whereas 50% of patients with elevated ALT had a normal liver on patohistological examination [28]. Similar concerns are also present in children [26,27]. However, according to a great need for screening the only widely accessible method is to determine ALT level (two times the age-specific upper normal level) in at-risk populations: overweight children, and those with diabetes type 2 and dyslipidemias [26]. The optimal age to screen has not been established yet, but the importance of lifestyle interventions suggests that early screening is warranted.

On some occasions, NAFLD can be found accidentally when performing diagnostics for, e.g., recurrent abdominal pain. Regardless of symptoms which are not present in NAFLD until the late stages, if increased liver transaminases or hyperechogenic liver by abdominal ultrasound are found, complete diagnostics have to be performed [26].

#### 3.3.2. Laboratory

The single most evaluated and commonly used test is ALT, but it is not specific, so other liver diseases with similar presentation have to be ruled out. Normal values are age- and gender-specific. ALT increased to more than twice the upper level after the exclusion of other causes in overweight children older than 10 years strongly indicates for NAFLD (88% sensitivity and 26% specificity) [26]. 

Recently, it was found that ALT and GGT correlate well with liver biopsy in children with NASH. The analyzed data were from another clinical trial in which the treatment with cysteamine bitartrate was studied. In a group of 146 children who had liver chemistry and liver biopsy performed at the beginning of the study and after 52 weeks, ALT and GGT significantly improved in a subgroup with liver histology improvement [29].

Laboratory panels for the screening of NAFLD or to determine its progression to NASH are searched for, but for now no laboratory test combination is included in the latest published international guidelines [26]. In a study of 222 children and adolescents with and 337 without NAFLD with the method of high-resolution metabolomics (liquid chromatography with ultra-high resolution mass spectrometry) measuring, thousands of small molecule metabolites in plasma 11 were identified, which achieved a sensitivity of 73% and specificity of 84% for NAFLD [30]. A different type of laboratory testing of lipid metabolites in plasma in 21 children with NAFLD and 21 without revealed significant differences in five lipid classes which might someday serve as a screening test [31]. Another possible biomarker for NAFLD is increased fibroblast growth factor 21, as was shown by meta-analysis, but there is not enough evidence for now to include it in routine screening [32]. 

A major part of laboratory workup serves to rule out other diseases with a similar presentation. The scope of investigations depends on the age, history, physical examination, and risk factors for NAFLD and comprises infections and autoimmune, genetic, and endocrine causes [26]. 

#### 3.3.3. Imaging

Abdominal ultrasound is not sensitive enough for the detection of lower degrees of liver steatosis (involving less than 33% of hepatocytes), but is a useful and widely available diagnostic method to rule out other causes of liver disease. Imaging methods to measure liver fibrosis are transient elastography, share wave elastography, and magnetic resonance elastography [29]. 

Transient elastography is a valuable and reproducible ultrasound-based method to measure liver stiffness (LSM; a surrogate for hepatic fibrosis) and controlled attenuation parameter (CAP; a surrogate for liver steatosis). Intra- and inter-operator agreement was assessed in 34 children. Agreement was better in intra- then in inter-observer liver stiffness measurement (concordance correlation coefficients of 0.85 and 0.76, respectively) than in intra- and inter-observer liver steatosis (concordance correlation coefficients of 0.73 and 0.58, respectively) [33]. 

Two newer sonographic methods for fibrosis detection, point and two-dimensional share wave elastography, have been shown to have low levels of technical failure and have been comparable [34]. The same meta-analysis reported that transient elastography was unreliable in 12.1% of measurements in children and adolescents.

Magnetic resonance elastography, another noninvasive method to assess liver fibrosis showed a correlation with the following histopathologic features: fibrosis and ballooning, as shown in a group of 50 adolescents [35]. In the same study, similar results were obtained with time-harmonic elastography based on ultrasound. 

#### 3.3.4. Liver Biopsy

Liver biopsy is the current standard for the diagnosis of NAFLD and to assess the severity, i.e., the presence of hepatic steatosis, inflammation, and fibrosis, and to rule out other diagnoses if the sample is representative (its length should be at least 2 cm). In extremely obese children (BMI more than 120th percentile or more than 35 kg/m^2^), liver biopsy is more difficult to perform due to a thicker subcutaneous tissue layer [26]. 

A semiquantitative liver histology scoring system for NAFLD was validated in adults and in children. Steatosis (less than 5% of steatotic hepatocytes is considered normal), lobular inflammation, hepatocellular ballooning, and fibrosis are assessed [36]. 

Liver biopsy is not uniformly performed in all children with NAFLD due to its invasiveness [9]. However, it must be performed at least in children with unclear diagnosis, in those with normal weight (“lean NAFLD”), or in those with increased risk or signs of advanced fibrosis [26]. The presence of NASH or advanced fibrosis should lead to even more decisive measures to reduce hepatic steatosis and consequent inflammation.

### 3.4. Treatment

For now, there is no single evidence-based treatment for NAFLD in children, apart from lifestyle modification which causes weight loss. Some improvements of different aspects of NAFLD were seen after specific interventions, e.g., antioxidants, polyunsaturated fatty acids or probiotic supplementation, but there is not enough evidence to support these treatments universally. One of the problems in published studies is heterogeneity of outcome measures. In a recently published systemic review, only 14% of pediatric studies used paired histology as an outcome measure, and 62% of included children did not have a liver biopsy to diagnose NAFLD, most likely due to the invasiveness [37]. 

Lifestyle interventions are currently the only successful treatment for NAFLD in children, but it is difficult to maintain the reduced body weight in the long run, as was shown in a study of 79 obese post-pubertal adolescents with a BMI of more than the 95th percentile for their age. They were randomized into two groups, to the lifestyle modification program (weekly counseling sessions for 4 months, then bimonthly for 52 weeks) which consisted of knowledge, attitude, and practice regarding diet and exercise based on motivational interview and behavioral modification, and to the control group, in which diet and exercise advice were provided during routine consultations every 4 months. Anthropometrics, laboratory results and intra-hepatic triglyceride content (assessed by magnetic resonance spectroscopy) were measured at baseline, week 16, and week 68. The improvement was significantly better in the intervention group after 16 weeks of intervention (body weight, body mass index, body fat, AST/ALT ratio, fasting insulin), but at 68 weeks children in the intervention group regained some weight and there was no difference any more between the intervention and control group [38]. 

Lifestyle interventions and a psychoeducation program was not enough to reduce hepatic fat as measured by magnetic resonance, as was shown in a study of 102 overweight or obese children aged 10.6 ± 1.1 year. The liver fat content significantly decreased only in the intervention group which had, in addition to the above mentioned lifestyle intervention and psychoeducation, supervised intense aerobic workouts three times weekly for 90 min. Similar improvements were also seen in laboratory metabolic and liver tests [39]. 

Specific interventions for the treatment of obesity and NAFLD have to be integrated with cognitive behavioral techniques and should include the whole family to be more successful. Intervention strategies have to be community based to adapt them to the possibilities of the patient’s environment [40]. 

#### 3.4.1. Physical Activity

Physical activity in children with NAFLD as the only intervention is only recently in the focus of research. Beforehand, it has been studied only in connection with other lifestyle interventions [37,41]. 

A systematic review and meta-analysis of the studies with supervised exercise training identified 16 studies published before January 2017. Fat content was measured with magnetic resonance or sonographically, and the types, intensity, volume, frequency, and duration of exercise varied. The study showed that isolated aerobic or resistance exercise trainings of equal to or more than 60 min, and a frequency equal to or more than three times per week reduced liver fat content and improved cardiovascular fitness and muscular strength [42]. 

In a randomized controlled trial (RCT) of 102 overweight or obese children, the intervention included three supervised exercise sessions per week, while both the intervention and control groups had 11 sessions of family-based lifestyle and psycho-educational programs. Hepatic fat assessed by magnetic resonance imaging reduced in significantly more patients in the intervention than in the control group (54% vs. 34%), as did weight, BMI, and GGT [43].

In another RCT, 107 adolescents with a BMI of 34.7 ± 4.1 kg/m^2^ were randomly assigned to a high-intensity or low-intensity training 12-week intervention and a control group. In contrast to the control group, high- and low-intensity training groups also received nutritional, psychological, and clinical counseling. Some NAFLD markers improved in both intervention groups (high-density lipoprotein, ALT, AST), but there was no change in glucose, insulin, and insulin resistance. Hepatic fat has not been assessed [44].

In contrary, an intense short-term (1 week) physical activity intervention together with reduced caloric intake in 57 obese children (age 12.0 ± 0.8 years, BMI 26.5 ± 3.2 kg/m^2^) led to an increased liver fat content assessed by computer tomography scans at the beginning and after seven days, which was probably attributed to the weight loss (−2.5 ± 0.9 kg) and decrease in BMI (−1.2 ± 0.4 kg/m^2^). ALT did not change while AST significantly increased [45]. 

#### 3.4.2. Dietary Modifications

Diet is a risk factor for pediatric NAFLD, and dietary intervention is one of cornerstones of NAFLD treatment. In a recently published systematic review [16], despite inconclusive results, the most important step to improve liver outcomes was weight loss, regardless of being achieved by low-carbohydrate or by low-fat diets. Another important observation was the improvement of liver parameters (a reduction in hepatic steatosis, ALT, and AST). 

A Mediterranean diet, which is low in saturated fats and animal proteins and high in antioxidants, fiber, and monounsaturated fatty acids might treat NAFLD in children, but the evidence is scarce [46]. Anyway, it is a healthy diet option preventing obesity and NAFLD development [47].

We could not find any data on a ketogenic diet for the treatment or prevention of NAFLD in children.

Not only overall caloric intake, but also specific nutrients can influence liver fat content, as was shown in a randomized controlled study of 40 adolescents with histologically diagnosed NAFLD [48]. Free sugar intake (glucose, fructose, and sucrose) was restricted to less than 3% of daily calories for 8 weeks. Liver fat content was measured by magnetic resonance imaging proton density fat fraction and reduced from 25% to 17% in the study group, and in the control group it was similar (21% to 20%). A significant decrease in ALT was detected in the study group. The data on overall caloric intake of both groups have not been collected.

Our search identified two more studies of NAFLD in children and nutrition published after 31 May 2020 when the literature search for previously mentioned systematic reviews [16] had finished.

The first study measured the hepatic fat fraction with magnetic resonance, ALT, and some metabolic parameters at the beginning and after 12 weeks of a normocaloric diet with a low ratio of n-6 to n-3 poly-unsaturated fatty acids [49]. In 17 obese adolescents, the hepatic fat fraction decreased by 25.8% and ALT reduced by 34.4% without a reduction in body weight. 

In the second study, authors assessed dairy fat intake, plasma fatty acids, and hepatic steatosis measured by magnetic resonance in 237 children aged 8 to 17 years [50]. An inverse correlation of diary fat intake and hepatic steatosis was found and is possibly associated with an effect of pentadecanoic acid, as has already been shown in some studies in adults. 

#### 3.4.3. Probiotics

Probiotics modulate the immune function of intestinal microbiota and by influencing the gut–liver axis, affecting hepatic metabolism. A recently published meta-analysis of RCTs in adults and children could not determine the effect of probiotics on NAFLD in children due to the heterogeneity of the groups [51].

In addition to the meta-analysis, there are only a few small studies on probiotics in pediatric NAFLD. On the other hand, in an RCT of 64 obese children aged 10 to 18 years, another mixture of probiotics (containing *Lactobacillus acidophilus* ATCC B3208, *Bifidobacterium lactis* DSMZ 32269, *Bifidobacterium bifidum* ATCC SD6576, and *Lactobacillus rhamnosus* DSMZ 21690) improved ALT, AST, cholesterol, low-density lipoprotein-C, and triglycerides levels, caused waist circumference decrease, and improved liver sonography results without influencing weight and BMI [52].

In an RCT of 22 children with biopsy-proven NAFLD treated with a mixture of probiotics (*B. longum* Y10, *B. infantis* Y1, *B. breve* Y8, *L. acidophilus*, *L. casei*, *L. del-brueckii* subsp. bulgaricus, *L. plantarum*, and *Streptococcus salivarius* subsp. thermophilus), liver steatosis improved after 4 months of treatment assessed by ultrasonography [53]. However, in another small placebo-controlled study of 19 obese adolescents with the same mixture of probiotics, those in the study group even gained weight and had no measurable differences in other parameters (liver fat content assessed by magnetic resonance, insulin and glucose concentrations, and gut microbiome) [54].

In conclusion, gut microbiome modulation is a promising treatment or prevention target for NAFLD in children, but larger and longer lasting studies are needed. Recent advances in omics platforms might enable the development of personalized and optimized probiotics for different conditions, including NAFLD treatment [55]. 

#### 3.4.4. Medical Treatment and Nutritional Supplements 

A recently published systematic review reports on 4 studies on treatment with metformin in children with NAFLD. Metformin-improved insulin resistance was in some studies associated with reduced steatosis on ultrasound, and had a potential for histological improvement of ballooning degeneration in NASH [37]. In another review, conflicting results of the studies were found regarding metformin treatment of NAFLD [56]. It is recommended to treat insulin resistance but cannot be recommended for the targeted treatment of NAFLD.

The data on vitamin E were studied in six RCTs before January 2018, and conclusions of systematic reviews were limited due to the heterogeneity of the studies. Vitamin E did not show significant benefits over a placebo, but it improved some features of NASH. Despite its safety, there are insufficient data on its efficiency to promote its use in children with NAFLD [37].

Polyunsaturated fatty acids were studied in six studies in children with NAFLD before January 2018. They were safe and well tolerated. They did not influence ALT, but they improved liver steatosis (in four studies it was assessed by ultrasound echogenicity, and in one study by hepatic fat fraction measured by magnetic resonance imaging) [37]. Omega-3 fatty acids alone or in combination with vitamin E or D showed promising results in some studies, but the evidence is insufficient to uniformly introduce those treatments [56].

Cysteamine is a precursor amino acid for the synthesis of glutathione which is a major intracellular antioxidant in liver. In a multicenter RCT of twice-daily treatment with cysteamine bitartrate, delayed release was compared to a placebo in 169 children with histologically determined NAFLD with the activity score of 4 or higher. The intervention lasted for one year. In the primary outcome, the improvement of liver histology, there was no statistical difference between groups. However, ALT, AST, and lobular inflammation decreased significantly in the treated group [37,57]. 

Choline, ursodeoxycholic acid, and other nutritional supplements or medications have been studied, but, for now, without a convincing influence on NAFLD [56]. Genetic background can play a role for some interventions, as has been shown for docosahexaenoic acid and patients with specific genetic variant (I148M PNPLA3) [56].

#### 3.4.5. Bariatric Procedures

Bariatric surgery is an effective treatment together with other measures in selected adults with morbid obesity [58]. Due to the possible adverse effects of specific deficiencies due to bariatric surgery on linear growth in children, bariatric surgery is indicated in morbidly obese adolescents after growth completion, and only as a part of multidisciplinary treatment. Mostly, two different surgical techniques also with good results in adolescents are performed: a Roux-en-Y gastric bypass, and a reversible adjustable gastric band placement. After the first procedure, patients have to be monitored to prevent micronutrient deficiencies. Gastric banding is similarly efficient and safer; therefore, it is gaining more popularity [58].

In a study of 11 morbidly obese (BMI between 40 and 50 kg/m^2^) and seven superobese (BMI more than 50 kg/m^2^) adolescents 18 months after gastric banding, weight loss was similar: 30 ± 19 kg and 28 ± 12 kg, respectively. Investigations for NAFLD (ALT, AST, and intraoperative liver biopsy) were performed before the gastric band placement only so an influence of NAFLD parameters was not assessed. Resolution of insulin resistance and dyslipidemia was greater in the morbidly obese group [59].

An endoscopically placed duodenojejunal bypass liner is a reversible bariatric procedure. In a study of 19 post-pubertal morbidly obese adolescents with at least one co-morbidity due to the obesity one year after the intervention, the decrease in weight loss was 11.4% (7.4 to 15.3%), and BMI decreased from 41.1 kg/m^2^ (40.1 to 44.1 kg/m^2^) to 37.2 kg/m^2^ (35.2 to 39.2 kg/m^2^). Liver steatosis improved in 68.4% and totally reversed in 52.6%. The ALT level dropped concomitantly [60].

### 3.5. Prevention

The prevention of NAFLD in children is similar to the prevention of obesity and consists of a healthy lifestyle, including a healthy diet (a Mediterranean diet being one of the possibilities), avoiding high intake of free sugars, physical activity, adequate sleep, and psychosocial well-being [15,40,61]. 

### 3.6. Prognosis

The NAFLD may proceed to liver cirrhosis even in the child age [2]. Hepatocellular carcinoma due to NAFLD is very rare, with only one child reported with this at the age of 7 years. Hepatocellular carcinoma has developed in severely steatotic liver and extensive differential diagnostics did not reveal any alternative diagnosis [62]. In a retrospective longitudinal cohort study of 66 children with NAFLD identified from 1985, after the exclusion of other liver diseases, and after a mean follow-up of 6.4 years (standard deviation 4.5 years, range 0.05 to 20 years), two patients underwent liver transplantation due to the end-stage liver disease due to NASH [63].

## 4. Discussion

In this review we summarized up-to-date information on NAFLD in children to provide information on recent advances and approaches to the prevention, diagnosis, and treatment of pediatric patients with NAFLD for a clinician who is not deeply involved in the field. 

In the discussion, special emphases are made on the practical application of the current knowledge. 

Despite broad knowledge of pathogenic mechanisms leading to overweight and obesity, which are major risk factors for NAFLD, the prevalence of this insidious disease is increasing in children and adolescents and has become the most common cause of liver disease in children [24,25]. 

There are several causes for the increased prevalence of NAFLD in children:

1. The disease is asymptomatic until the progression to end-stage liver disease that happens after many years or even decades [64].
2. The treatment is lifelong lifestyle modification with a stress on healthy diet and physical activity, which is difficult to maintain [26].
3. Lifestyle which leads to obesity/overweight and NAFLD is usually not an isolated problem of specific patient, but is mostly not only medical, but also a psychosocial problem of the whole family, so treatment interventions have to be multidisciplinary, multifaceted, and long-lasting, which is difficult to achieve [40].
4. Free sugars and fats are added to ready-made foods to make them more palatable and to increase consumption and sales. Human taste is prone to give preference to sweet and energy dense foods [65].
5. Public health authorities can rarely oppose the beverage and food industry which maintain high sugar and other inexpensive ingredient content, which promote sales but are harmful to health [66].

Prevention is the most important to reduce NAFLD in children, and consequently in adults. Programs must extend beyond addressing already obese children by pediatrician and nutritionist and must include families and community [40]. They should start early enough, ideally in the prenatal period [3].

NAFLD must be actively searched for in children with increased risk, i.e., in overweight or obese children, or in those with dyslipidemias, insulin resistance, or diabetes mellitus type 2. There is no ideal test for screening yet, so ALT determination is used as the first test and it should start at the age of 9 years [26]. 

Diagnosis is made by proving hepatic steatosis and the exclusion of other diseases which also present with fatty liver. The extent of diagnostic testing depends on the presentation and age. In an infant or young child with NAFLD, it is broader than in an adolescent with typical risk factors for NAFLD [6]. Different sets of tests are listed in Table 1.

Fibrosis can be assessed and followed up by different modalities of ultrasound elastography or magnetic resonance elastography, with ultrasound methods being usually more accessible [26]. 

Liver biopsy should be performed in those with unclear diagnosis or in children who have increased risk or signs of advanced fibrosis, splenomegaly, two to three times higher ALT despite lifestyle modification and some weight loss, and an increased ratio of AST/ALT > 1 [26].

Regarding follow-up, there are no unique guidelines. Usually, children are monitored every year with at least ALT and, if accessible, with some imaging techniques to estimate the level of fibrosis in the liver [27].

The goal of treatment is the regression of liver steatosis, inflammation, and fibrosis. At present, the only effective treatment of NAFLD in children is lifestyle modification which consists of dietary intervention and increased physical activity [26]. In the case of insulin resistance, treatment with metformin is recommended [37]. 

To achieve long-term success, an integrative approach, including cognitive-behavioral techniques, family- and community-based interventions, and tele-health should be implemented [40].

## 5. Conclusions

NAFLD in children is a big medical and public health challenge due to high and increasing prevalence and is a great influence on a child health and prognosis when they become adults. NAFLD is not an isolated disease, but is a part of a metabolic syndrome in the majority of patients; therefore, it has to be addressed by a multidisciplinary team of experts to prevent complications and improve overall health of the population.

## Figures and Tables

**Table 1:** Different sets of tests in children with suspected NAFLD according to the age and presentation.

Basic Laboratory Profile	Metabolic Function	Tests for Exclusion of Other Main Causes of Hepatic Steatosis	Advanced Set of Tests in Children Younger Than 10 Years or Atypical Presentation
blood count standard liver function tests with ALT/AST urea and electrolytes coagulation with INR	fasting glucose and insulin ammonia lipid profile glucose tolerance test thyroid function tests	serum iron and ferritin serum cooper, ceruloplasmin sweat test celiac disease screening a1-antitripsin level and phenotype viral hepatitis panel serum immunoglobulins and liver autoantibodies	serum lactate, uric acid, pyruvate amino acids in plasma organic acids in urine acyl carnitine profile

## Data Availability

Not applicable.
